# *Mycobacterium microti*: Not Just a Coincidental Pathogen for Cats

**DOI:** 10.3389/fvets.2020.590037

**Published:** 2020-12-03

**Authors:** Sophie Peterhans, Patricia Landolt, Ute Friedel, Francisca Oberhänsli, Matthias Dennler, Barbara Willi, Mirjam Senn, Sandro Hinden, Karin Kull, Anja Kipar, Roger Stephan, Giovanni Ghielmetti

**Affiliations:** ^1^Section of Veterinary Bacteriology, Vetsuisse Faculty, Institute for Food Safety and Hygiene, University of Zurich, Zurich, Switzerland; ^2^Kleintierpraxis Dr. med.vet. F. Oberhänsli, Märstetten, Switzerland; ^3^Clinic for Diagnostic Imaging, Department of Clinical Diagnostics and Services, Vetsuisse Faculty, University of Zurich, Zurich, Switzerland; ^4^Clinic for Small Animal Internal Medicine, Vetsuisse Faculty, University of Zurich, Zurich, Switzerland; ^5^Tierklinik Thun, Thun, Switzerland; ^6^Dres. Kull, Gross- und Kleintierpraxis, Ernen, Switzerland; ^7^Vetsuisse Faculty, Institute of Veterinary Pathology, University of Zurich, Zurich, Switzerland

**Keywords:** *Mycobacterium microti*, *Mycobacterium tuberculosis* complex, vole bacillus, pyogranulomatous lymphadentitis, nontuberculous mycobacteria, interferon-gamma assay

## Abstract

Public interest in animal tuberculosis is mainly focused on prevention and eradication of bovine tuberculosis in cattle and wildlife. In cattle, immunodiagnostic tests such as the tuberculin skin test or the interferon gamma (IFN-γ) assay have been established and are commercially available. Feline tuberculosis is rather unknown, and the available diagnostic tools are limited. However, infections with *Mycobacterium tuberculosis* complex members need to be considered an aetiological differential diagnosis in cats with granulomatous lymphadenopathy or skin nodules and, due to the zoonotic potential, a time-efficient and accurate diagnostic approach is required. The present study describes 11 independent cases of *Mycobacterium microti* infection in domestic cats in Switzerland. For three cases, clinical presentation, diagnostic imaging, bacteriological results, immunodiagnostic testing, and pathological features are reported. An adapted feline IFN-γ release assay was successfully applied in two cases and appears to be a promising tool for the ante mortem diagnosis of tuberculosis in cats. Direct contact with *M. microti* reservoir hosts was suspected to be the origin of infection in all three cases. However, there was no evidence of *M. microti* infection in 346 trapped wild mice from a presumptive endemic region. Therefore, the source and modalities of infection in cats in Switzerland remain to be further elucidated.

## Introduction

Tuberculosis (TB) remains one of the most important infectious diseases of humans worldwide, with about one third of the world's population carrying the bacteria and 1.5 million deaths in 2018 (1). Bovine tuberculosis (bTB), caused by *Mycobacterium* (*M*.) *bovis*, and to a lesser extent by *M. caprae*, is endemic in animal populations in many parts of the world and in 2016 was responsible for an estimated 147,000 cases of zoonotic TB, mainly in the African and South-East Asian regions (1, 2). In the past decades, the stringent test-and-cull policies applied in western countries significantly reduced the bTB incidence (3). However, wildlife reservoirs play an important and sometimes unpredictable role in disease transmission to farm animals, e.g., badgers in the UK (4), possums in New Zealand (5), or wild boars in Spain (6). The control of such animal reservoirs can directly influence the prevalence of human TB, as seen in New Zealand (7, 8) or the UK (9).

The field vole is considered the maintenance host of *M*. *microti* (10, 11), a member of the *M. tuberculosis* complex (MTBC). The bacterium has pathogenic potential in a broad range of species, as indicated by reports on natural infections in free-ranging wild species [wood mice, shrews (12), badgers (4) and wild boars (13, 14)], captive wild animals [new world camelids (15, 16), meerkats (17), gibbons (unpublished data) and squirrel monkeys (18)], livestock species [cattle (19, 20), pigs (21) and goats (22)], pet animals [cats (23) and dogs (24)], and humans (25, 26). The full pathogenic potential of *M. microti* in humans is not yet defined, but since it carries a deletion in the region of difference 1 (RD1), an important genomic locus coding for well-known virulence factors, it is likely less virulent and pathogenic than other members of the MTBC (27). However, pulmonary and disseminated disease due to *M. microti* have been described in both immunocompromised and immunocompetent patients in different European countries (25, 26, 28–30). Recently, six cases of pulmonary tuberculosis due to *M. microti* were described in France (26), suggesting that the pathogenic potential of the bacterium is higher than previously estimated. Interestingly, *M. microti* seems to be geographically restricted to continental Europe and the UK since so far, no reports of *M. microti* infections in animals or humans have been published elsewhere. Also, different from the UK, where field voles have been identified as the maintenance host of the bacteria (10), the reservoirs in continental Europe are still unknown despite various investigations (13). Studies in the UK provided strong evidence that free-ranging cats contract *M. microti* through hunting of infected field voles in certain areas, whereas they become infected with *M. bovis* in other geographical regions (31). By now, feline tuberculosis is considered an emerging disease (11, 32). Depending on the entry site of the mycobacteria, the clinical presentation can be extremely variable. Cutaneous lesions resulting from fighting and biting with lymphadenitis are most frequent, but gastrointestinal or respiratory signs and, rarely, joint involvement is reported (32). In Switzerland, a first case of *M. microti* infection in a cat was reported in 2011 (23).

The diagnosis of tuberculosis in companion animals is challenging due to limited *in vivo* diagnostic options. The collection of biopsy material for bacteriology and histopathological examination is difficult and invasive depending on the location. A general anesthesia is often necessary, and owners might be reluctant to take further diagnostic steps due to the costs and risk related to such procedures. Culture-based detection is still the gold-standard for MTBC members, however, this procedure requires weeks for an aetiological diagnosis. More recently, new molecular methods to detect MTBC members directly from tissue samples, based on the *mpb70* gene and single nucleotide polymorphisms within *gyrB* and *gyrA*, were developed (33–35). These simplify the diagnostic approach and the latter may allow differentiation between *M. bovis* and *M. microti*, the main aetiological agents of feline TB within a few hours. Moreover, an immunological test for indirect detection of TB infected cats is available. This assay is based on testing peripheral blood mononuclear cell (PBMC) antigen-specific interferon (IFN)-γ responses. More in detail, a subpopulation of T cells from an animal sensitized to a MTBC member, induce a type IV hypersensitivity reaction. This cell mediated reaction can be recognized through the measurement of IFN-γ produced within a given incubation period and has a good predictive capacity for both pathogens (32, 36).

The present study was prompted by the occurrence of 11 feline tuberculosis cases caused by *M. microti* in Switzerland between 2014 and 2019. Thus, the aims of this study were (i) to provide the full work-up of three cases of *M. microti* infection in cats, including clinical presentation, diagnostic and therapeutic approaches; (ii) to identify potential maintenance hosts among four different wild rodent species in a Swiss *M. microti*-endemic area, where infected domestic and wild animals had previously been identified, and (iii) to evaluate the performance of a feline interferon-gamma-release assay (IGRA) based on culture confirmed cats.

## Materials and Methods

### Case Material

The study was undertaken on 11 natural feline TB cases (Tables 1A,B), all adult Shorthair cats. For three cases, all anamnestic and clinical data was available (Table 1A; cases 1–3). *Case 1*, euthanized after a long clinical history and previously confirmed *M. microti* infection, underwent partial post mortem examination with the owner's consent at the referring veterinary clinic; lung, liver, spleen, and body lymph nodes (mandibular, mesenteric, ileocolic, and popliteal lymph nodes) were submitted fresh for bacteriological and histopathological examination. *Case 2*, was euthanized and submitted for diagnostic post mortem examination with the owner's consent. Samples of lung, mandibular and popliteal lymph nodes were subjected to a bacteriological examination. All major organs were sampled for histological examination. From *Case 3*, surgical biopsies from skin lesions and mandibular lymph node were submitted for bacterial culture and histopathological examination. For the remaining eight cases (cases 4–11), anamnestic data was missing, and information on the clinical signs were limited; however their geographical origin was known (Table 1B).

**Table 1A:** Overview of the diagnostic workup of the three cases of feline tuberculosis.

**Case**	**Geographical origin**	**Symptoms/lesions**	**Radiology**	**FNA**	**Histopathology**	**Bacteriology**	**IGRA (BoPPD/ AvPPD)**
1	Märstetten, Thurgau	Generalized enlarged LN, multiple subdermal skin nodules	n/a	LN: numerous (60%) mature lymphocytes and moderate (20%) neutrophils partly degenerated	Skin nodule: high degree pyogranulomatous panniculitis; LN: lymphadenitis, no AFB by ZN staining	AFB in ZN staining, positive MTBC real-time PCR, *M. microti* in culture (6 weeks)	0.50/0.246
2	Wolfhausen, Zurich, originally from eastern Europe	Enlarged mandibular LN, respiratory symptoms	Radiography: severe increase of pulmonary opacity, severe bronchial-interstitial pattern; CT: severe, generalized increase in pulmonary attenuation	LN: activated macrophages indicating a pyogranulomatous inflammation (low cellularity of the smear)	LN and lung: granulomatous inflammation with intralesional AFB	Curved AFB in ZN staining, positive MTBC real-time PCR, *M. microti* in culture (4 weeks)	n/a
3	Fieschertal, Valais	Enlarged mandibular LN, ulcerative skin lesions	CT: severe mandibular and retropharyngeal lymphadenopathy, miliary pulmonary pattern	LN: numerous lymphatic cells, mainly small lymphocytes and moderate (10–20%) neutrophils without degeneration	LN: massive chronic granulomatous to pyogranulomatous lymphadenitis, no AFB by ZN staining	Curved AFB in ZN staining, positive MTBC real-time PCR, bacterial species identification by HRM, no growth of *M. microti*	7.82/2.96

*LN, lymph nodes; AFB, acid fast bacilli*.

**Table 1B:** Overview of the additional eight cases of feline tuberculosis caused by *Mycobacterium microti* diagnosed in Switzerland between 2013 and 2017.

**Animal data/isolation**	**Geographical origin**	**Clinical signs and findings**	**Relevant histopathological findings**	**Outcome**
2 y, FN, 2013	Seftigen, Bern	Severe dyspnea, weight loss; radiography: diffuse increase of pulmonary opacity	Lung and bronchial LN: granulomatous inflammation with large quantities of AFB in macrophages	Euthanized
2014	Oberbütschel, Bern	Severe dyspnea	NA	NA
7 y, FN, 2014	Chernex, Vaud	Enlarged mandibular LN	Granulomatous lymphadenitis with AFB in macrophages	NA
5 y, MN, 2015	Plasselb, Fribourg	Severe dyspnea and massive weight loss	Granulomatous pneumonia with AFB in macrophages	NA
5 y, MN, 2015	Plasselb, Fribourg	Enlarged mandibular LN; Generalized subcutaneous nodular lesions; radiography: miliary pulmonary pattern	Granulomatous lymphadenitis with AFB; granulomatous panniculitis without AFB; granulomatous pneumonia without AFB	Euthanized
F, 2015	Zurich, Zurich	Dyspnea, no response to treatment; radiography: diffuse increase in pulmonary opacity	Granulomatous pneumonia with rare AFB in macrophages	Euthanized
9 y, MN, 2016	Attelwil, Aargau	Dyspnea, subcutaneous nodules, weight loss; radiography: multifocal pulmonary opacity	Lung, bronchial LN: granulomatous inflammation with AFB in macrophages; granulomatous dermatitis with AFB in macrophages	Euthanized
16 y, F, 2017	Roche, Vaud	Enlarged mandibular LN; Generalized subcutaneous nodular lesions	Granulomatous lymphadenitis with scarce AFB in macrophages; granulomatous panniculitis	Euthanized

*F, female; FN, female neutered; M, male; MN, male neutered; LN, lymph node; AFB, acid fast bacilli; NA, information not available; y, years*.

### Mycobacterial Analyses

#### Direct Detection: Bacteriology and Molecular Diagnosis

Clinical biopsies and post mortem samples were processed following a standardized protocol as described before (37). Briefly, the samples were homogenized and decontaminated using H_2_SO_4_ (4%), followed by neutralization with NaOH (1 M). Further methods included direct Ziehl Neelsen (ZN) staining, MTBC real-time PCR targeting the potentially multi-copy IS*6110* gene (38), spoligotyping using a commercial microarray system with integrated data analysis (Alere Technologies, Jena, Germany) (30), and a HRM assay based on single nucleotide polymorphisms within *gyrA* and *gyrB* (33, 34) for species identification. Because of the well-described fastidious and slow growth rate of the pathogen involved (25, 29, 39), cultures on liquid and solid media were incubated until growth of the mycobacteria was detected or for a maximum period of 12 months.

#### Indirect Detection: Interferon-Gamma-Release Assay (IGRA)

The performance of an adapted feline IGRA was evaluated based on two culture confirmed *M. microti* cats and 12 healthy cats with no history of MTBC infections in their household. Blood samples from the control cats were collected when available, with the owner's consent in cases where blood samples were taken for other purposes (blood donors, blood check for elective procedures, or annual health checks for aged cats). The blood samples were stimulated within 6 h after collection except for the sample originating from Case 3 where transport logistics only allowed stimulation 16 h after sampling. Previous studies have shown that feline whole blood, unlike bovine or human blood, does not respond well to antigen stimulation *in vitro* (36). Therefore, the PBMCs from heparinized whole blood were separated and diluted as previously described (36). The IFN-γ responses of the cell suspensions after stimulation with bovine and avian tuberculins (BoPPD and AvPPD; Bovigam TB kit, Thermo Fischer Scientific, Reinach, Switzerland) at a final dilution of 1:100 were assessed. A mitogen positive control (phorbol myristate acetate plus calcium ionophore (PMA/Ca) at 50 ng/ml and 1 mg/ml, respectively, Merk, Buchs, Switzerland) and two unstimulated aliquots [phosphate-buffered saline (PBS) and culture medium] were used as controls. Culture medium was prepared as previously described (36). Samples were incubated 4 days at 37°C, with 5% CO_2_ atmosphere on 96 F-bottom plates. After a centrifugation step of 10 min at 500 × g, cell-free supernatants were harvested and immediately assessed for the presence of IFN-γ with a commercial feline IFN-γ ELISA (RayBio Feline IFN-gamma ELISA Kit, Norcross, USA) or assessed after freezing at −20°C until evaluation. Standard feline IFN-γ provided by the manufacturer was recovered, diluted and measured in duplicates for each assay in accordance with the supplier's recommendations.

Stimulated supernatants and controls were analyzed in duplicates. The IFN-γ concentrations were calculated based on the mean optical density (OD) values measured at 450 nm. The 4-parameter logistic regression equation was chosen to extrapolate the concentrations from the curve generated with the OD values of the standard. A positive response to a given stimulant (BoPPD and AvPPD) was defined as an IFN-γ concentration greater than the mean IFN-γ concentration of the background (PBS stimulation) for each cat plus 3 standard deviations of the mean, as previously described (40). For a test to be considered interpretable, the positive control (PMA/Ca) response had to be positive based on the same criteria when compared to the background (PBS). The minimum detectable dose of feline IFN-γ was 0.24 ng/ml according to the manufacturer's instructions.

### Screening of Wild Rodents

Among the most common Cricetidae and Rodentia species occurring in Switzerland, field vole (*Microtus agrestis*), bank vole (*Myodes glareolus*), and *Apodemus* sp. (yellow-necked mouse and wood mouse) were identified as potential maintenance hosts because of their known susceptibility to *M. microti* infection (10, 12, 41). In addition, montane water voles (*Arvicola scherman*) and common voles (*Microtus arvalis*) which are widespread on pastures and open fields and are often preyed by cats were included in the investigation. Wild rodents were trapped between April and November 2017 in the Gantrisch Nature Park, a *M. microti*–endemic region where infected domestic animals (four cats, one llama) and a wild boar had previously been identified (unpublished data).

Based on the only studies published to date (10, 31, 42) the estimated prevalence of *M. microti* infection in wild rodents varies between 2 and 13% in regions where vole TB occurs. The studies cited were all conducted in the UK and under conditions that are not entirely identical to those of the present project. In some instances, only a single species (*M. agrestis*) was captured or only older animals were considered. For these reasons a lower prevalence (5%) was assumed in our study. To allow an estimate of the prevalence based on a combination of diagnostic tests including PCR, gross lesion inspection, histopathology and culture (100% specificity and 96% sensitivity), the necessary sample size was 77 animals per species, with a confidence level of 95%. *Apodemus flavicollis* and *A. sylvaticus* were regarded as one species, as these are phenotypically indistinguishable. Sample size estimation was performed using https://epitools.ausvet.com.au. A total of four woodland and field spots of ~1 km^2^ were chosen in proximity to the location where the infected cats and the llama had lived.

Un-baited Topcat traps (Andermatt Biocontrol, Switzerland) were used to trap montane water voles and common voles in open fields, whereas live traps (Longworth, Penlon Ltd., Abingdon, UK) baited with cereals, apples, peanut butter, and straw were used for bank voles, field voles, yellow-necked mice, and wood mice as previously described (43). Live traps were placed in woodland and trapped mice were visually inspected and subsequently euthanized by exposure to CO_2_ at a controlled fill rate of 20% of the chamber volume per minute. Euthanasia took place on site and the mice were transported cooled to the laboratory, where necropsy, gross examination, and sampling were performed. From all animals, liver, spleen, lungs, mandibular, and mesenteric lymph nodes were collected for histological examination and bacteriological culture.

For bacteriological culture, tissues of each three animals were pooled, homogenized, and decontaminated as described elsewhere (37), resulting in a total of 116 pools. A direct real-time PCR was performed on the pools. Cultures showing growth of colonies suspicious of mycobacteria were screened for acid fast bacilli (AFB) with ZN staining. Genomic DNA was extracted and tested with the same real-time PCR as above to confirm MTBC members.

### Histological Examination and Immunohistology

Tissues samples from cats 1–3 and from all rodents were fixed in 10% non-buffered formalin for ~48 h, trimmed and routinely paraffin wax embedded. Sections (3–4 μm) were prepared and stained with haematoxylin and eosin (HE).

In the feline cases, consecutive sections were subjected to a routine ZN stain to detect AFB. Immunohistology for the demonstration of monocytes and macrophages (Iba-1^+^), T cells (CD3^+^), and B cells (CD45R^+^) was performed, following previously published protocols (44, 45).

## Results

### Feline Cases

Between 2014 and 2019 a total of 11 natural cases of feline tuberculosis caused by *M. microti* and originating from seven different cantons in Switzerland were identified (Tables 1A,B). Information on the clinical course and specific signs were limited for many cases, but showed a recurrent pattern of clinical signs, i.e., enlarged mandibular lymph nodes, subcutaneous nodular lesions and dyspnea. These generally reflected the presence of pathological processes in lungs, lymph nodes, and/or skin. Information on the histopathological processes was available for 10 cases. These all represented granulomatous inflammatory lesions, i.e., a granulomatous pneumonia, lymphadenitis and/or dermatitis/panniculitis. In most cases (7/10), acid fast bacilli were identified within macrophages in the inflammatory processes (Tables 1A,B).

Three cases (cases 1–3) were available for a thorough work-up. These are described in detail in the following section.

#### Case 1

##### Clinical history (Figure 1A, Table 1A)

A male neutered European Shorthair cat was referred to a veterinary practice for recurrent generalized enlargement of peripheral lymph nodes. Enlargement of the right mandibular lymph node had already been recognized 2 years earlier. The cat was free ranging and lived in an eastern region of Switzerland, where *M. microti* infection was previously described in wild boars (46). At the time of diagnosis, the cat was 7 years old and presented with multiple subcutaneous nodules and enlarged mandibular, pre-scapular and popliteal lymph nodes, ranging in diameter from 1.8 to 3.5 cm. The lymph nodes were firm upon palpation and movable in the surrounding tissue. The cat was in good general condition. The animal was tested negative for Feline immunodeficiency virus (FIV) and feline leukemia virus (FeLV) (SNAP® FIV/FeLV Combo Test, Idexx, Bäch, Switzerland) infection. A fine needle aspirate from the right mandibular lymph node showed a mixed cell population with numerous mature lymphocytes (~60%) and moderate numbers (~20%) of partly degenerated neutrophils; scarce macrophages, plasma cells, and fibroblasts were also seen. A bacterial lymphadenitis was suspected and two 7-day courses of amoxicillin/clavulanic acid (20 mg/kg q12 h) were administered over 1 month. By the end of the therapy, the lymph nodes had roughly halved in size. Five months later the cat presented with reduced general condition and loss of appetite, accompanied by generalized lymph node enlargement. In particular, the mandibular lymph nodes were enlarged, with an approximate diameter of 3 cm. Additionally, multiple subdermal nodules were present. Histopathological examination of five biopsied nodules revealed a severe pyogranulomatous panniculitis and lymphadenitis. AFB were not detected (ZN stain) and a non-specific bacterial infection suspected. A second therapy, by subcutaneous administration of cefovecin (8 mg/kg), was attempted. The general condition of the patient remained stable and the size of the lymph nodes did not change substantially for a period of 19 months, after which the cat was presented due to multiple episodes of vomiting. Another biopsy was taken from the enlarged right mandibular lymph node. This again represented a pyogranulomatous inflammation, but this time AFB were detected, and MTBC infection was diagnosed by PCR. Based on the diagnosis of tuberculosis and the further deterioration of the cat's general condition, a 3-week therapy regimen with doxycyclin (5 mg/kg q12 h) was attempted. One month later, the cat was euthanized due to a lack of clinical improvement and poor quality of life. Prior to euthanasia, a blood sample was collected for IGRA.

**Figure 1:**
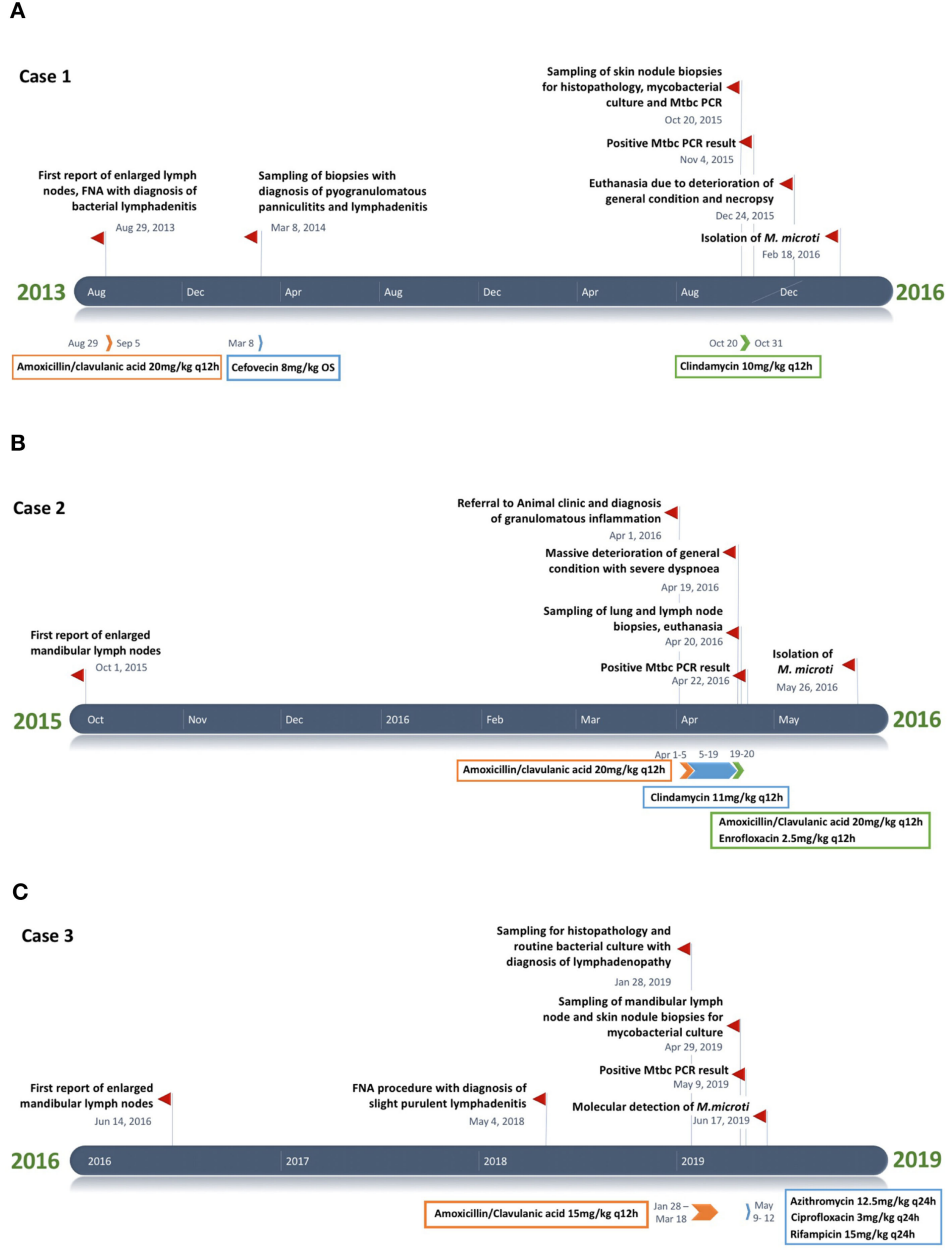
**(A–C)** Timeline chart presenting an overview of the clinical and therapeutic features of cases 1–3. The unspecific chronic symptoms and the diagnostic challenge of feline tuberculosis caused by *Mycobacterium microti* often result in a prolonged disease course.

##### Mycobacterial examination

Mandibular, popliteal, mesenteric and ileocaecal lymph nodes and lung collected at necropsy were subjected to mycobacterial culture. ZN staining of the homogenized tissue samples showed curved acid-fast bacilli. After 4–6 weeks of incubation, mycobacterial growth was yielded in liquid media cultures in all samples with the exception of the mesenteric lymph node. The colonies were identified as *M. microti* using spoligotyping.

##### Histopathological examination

**Figure 2:**
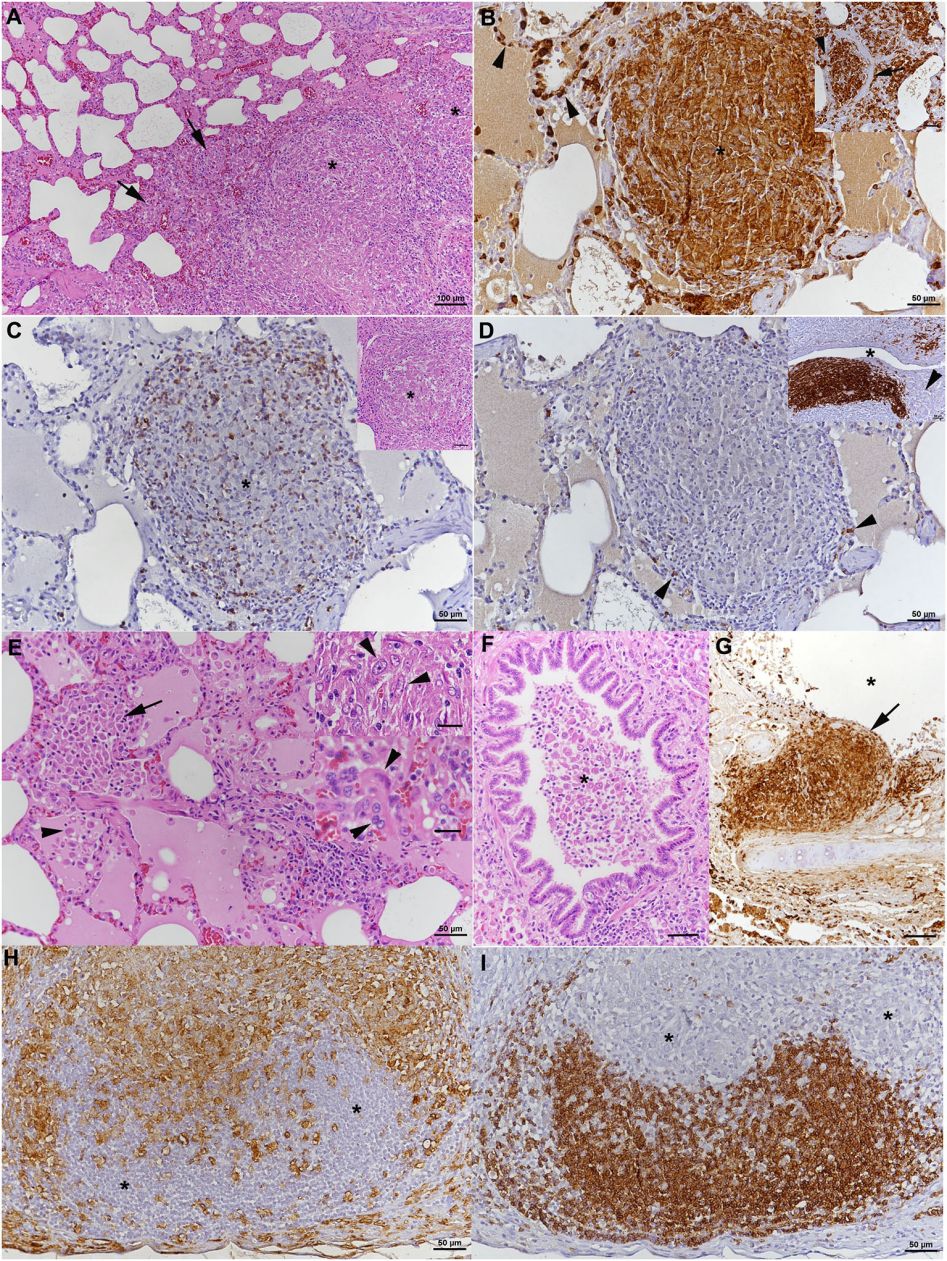
Case 1, histopathological features. **(A–G)** Lungs. **(A)** The parenchyma exhibits nodular, partly coalescing, non-demarcated granulomatous infiltrates (*) and alveoli that are packed with macrophages (arrows). HE stain. Bar = 100 μm. **(B)** The infiltrates (*) are dominated by Iba-1^+^ macrophages/epithelioid cells, and Iba-1^+^ macrophages are also confirmed to fill alveolar lumina (inset: arrows). Capillaries generally contain abundant monocytes (Iba-1^+^; arrows), providing evidence of monocyte recruitment into the tissue. Bars = 50 μm. **(C)** The granulomatous infiltrates (*) contain moderate numbers of disseminated T cells (CD3^+^). Inset: HE stain of infiltrate. **(D)** Only scattered B cells (CD45R^+^) are present in the infiltrate (arrowheads). However, they form lymphocyte aggregates close to bronchi (*: bronchiolar lumen), representing part of the bronchus associated lymphatic tissue (inset; arrowhead: bronchiolar glands). Bars = 50 μm. **(E)** Closer view of alveoli granulomatous infiltrates resembling accumulations of alveolar macrophages that fill and expand the alveoli (arrow). Adjacent alveolar lumen with edema fluid and individual desquamed alveolar macrophages (arrowhead). Within granulomatous infiltrates, there are abundant large epithelioid cells (top inset: arrowheads) and occasional bi- or tri-nucleated macrophages (bottom inset: arrowheads), but no multinucleated giant cells. HE stains. Bars = 50 μm. **(F)** Macrophages cells are also found in the exudate that fills the lumen of a small bronchiole (*). HE stain. Bar = 50 μm. **(G)** Bronchus with granulomatous infiltrate that breaks into the bronchial lumen (*; arrowhead). Immunohistology for Iba-1. Bar = 50 μm. **(H,I)** Mandibular lymph node. **(H)** Cortex and paracortex with effacement of the architecture my granulomatous infiltrates, as shown by the extensive infiltration of Iba-1^+^ macrophages that appear to invade the lymphatic follicles (*). **(I)** Immunohistology for CD45R confirms the cortical structures as follicles with adjacent granulomatous infiltrates (*). Bars = 50 μm.

Histopathological examination was performed on tissue samples from lung, liver, spleen, and the above listed lymph nodes. The lungs exhibited random multinodular, partly coalescing, non-demarcated granulomatous infiltrates (Figure 2A). The infiltrates were dominated by Iba-1^+^ macrophages/epithelioid cells (Figure 2B) and comprised small proportions of neutrophils and disseminated T cells (CD3^+^) (Figure 2C), whereas B cells (CD45R^+^) were sparse (Figure 2D). Multinucleated giant cells (MGCs) were not observed, but there were occasional bi- or tri-nucleated macrophages and abundant large epithelioid cells (Figure 2E). Multifocally, granulomatous infiltrates resembling accumulations of alveolar macrophages appeared to fill and expand alveoli (Figure 2E). Similar cells also formed the exudate that often filled the lumen of small bronchioles (Figure 2F). The granulomatous infiltrates were occasionally neighbored by areas of fibrosis or found to break into a bronchiole (Figure 2G). Capillaries generally contained abundant monocytes, and there was evidence of monocyte recruitment into the tissue (Figure 2B). In addition, B cell (CD45R^+^) dominated lymphocyte aggregates were found close to bronchi (part of bronchus associated lymphatic tissue, BALT; Figure 2D, inset) and randomly distributed. All lymph nodes exhibited similar changes, represented by variably sized granulomatous infiltrates that displaced the lymphatic tissue (Figures 2H,I). In mandibular and mesenteric lymph node, the lymph node architecture was extensively effaced and large areas of necrosis, without mineralization, were found within the granulomatous infiltrates (Figures 2H,I). The spleen also exhibited a small focal granulomatous infiltrate in the red pulp; the white pulp was composed of moderately sized secondary follicles and small T cell zones. The liver did not show any histological changes apart from scattered small aggregates of mononuclear cells in sinusoids. AFB were not detected in any of the tissues.

#### Case 2

##### Clinical history (Figure 1B, Table 1A)

**Figure 3:**
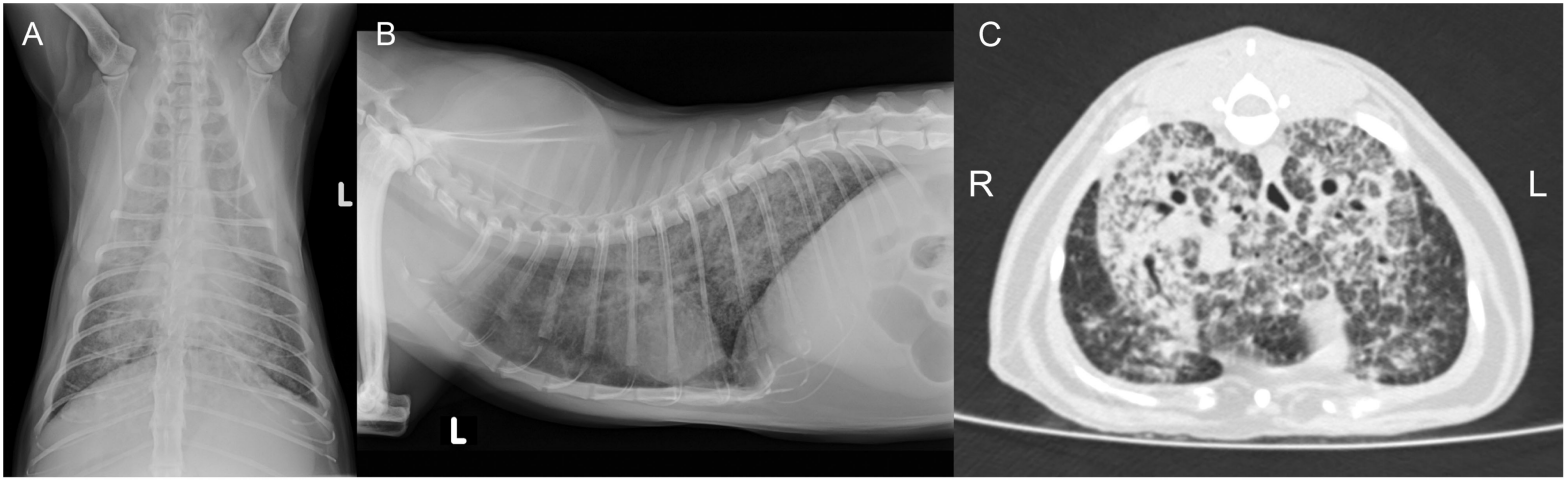
Case 2, diagnostic imaging. The thoracic radiographs in a ventrodorsal **(A)** and right-to-left lateral **(B)** projection reveal a generalized severe increase of pulmonary opacity with a heterogeneous distribution. The most severely affected areas show a broncho-interstitial toward an alveolar pattern. **(C)** Computed tomography of the thorax at the level of the cardiophrenic angle displayed in a lung window (Window level/Window width: −500/1,400 HU). There is a severe generalized increase in pulmonary attenuation with a mixed pattern, confluent to consolidated areas.

A 5-year-old free ranging male neutered Domestic Shorthair cat was referred to the Clinic for Small Animal Internal Medicine of the Vetsuisse Faculty, University of Zurich, due to tachypnea, dry cough, reduced appetite, and loss of weight. The private veterinarian had previously diagnosed an enlargement of the mandibular lymph nodes and a generalized interstitial lung pattern on thoracic radiographs. On presentation, the cat showed tachypnea (120/min) with inspiratory and expiratory increased lung sounds, and bilaterally enlarged and firm mandibular lymph nodes. Hematology revealed a slight neutrophil left shift, and serum biochemistry showed mild hyperproteinemia. The animal was tested negative for FIV and FeLV infection. Thoracic radiographs revealed a generalized, irregularly distributed increase of pulmonary opacity with a severe broncho-interstitial pattern coalescing to alveolar zones in the most severely affected areas (Figures 3A,B), consistent with severe bronchopneumonia. A fine-needle aspirate of the mandibular lymph nodes indicated a granulomatous inflammation. The cat was discharged with amoxicillin/clavulanic acid (20 mg/kg q12 h for 3 weeks) and imidacloprid (80 mg)/moxidectin (8 mg) spot on twice, 2 weeks apart. After 4 days the owners reported anorexia and vomitus, therefore the antimicrobial treatment was changed to clindamycin (11 mg/kg q12 h for 4 weeks). Three weeks after the first consultation the cat was reassessed at the clinic due to severe clinical deterioration and progressive dyspnea. On presentation the cat showed severe apathy, tachypnea (140/min) and dyspnea, bilateral generalized increased lung sounds, and slightly elevated body temperature (39.2°C); the mandibular lymph nodes were still enlarged and firm. Computed tomography was performed in general anesthesia and revealed severe generalized heterogeneous increase in attenuation of the lung with a mixed pattern, confluent to areas of consolidation in the most severely affected areas. The cranial mediastinal lymph node was mildly enlarged (7.5 mm in diameter) (Figure 3C). The findings were compatible with severe bronchopneumonia of bacterial, fungal, or parasitic origin, with inflammatory lymphadenopathy. After CT, the cat underwent surgery to collect incisional biopsies of the middle and caudal pulmonary lobes. After surgery, the cat was not able to breathe autonomously and was euthanized for welfare reasons. A full necropsy was performed.

##### Mycobacterial examination

Lung and lymph node specimens were subjected to standard bacteriological screening (aerobic and anaerobic culture), with negative results. Direct MTBC real-time PCR was positive. ZN staining of the decontaminated material showed curved AFB. Lung and mandibular, mesenteric and popliteal lymph node specimens were subjected to mycobacterial culture. After ~4 weeks, cultures yielded mycobacterial growth, and *M. microti* was confirmed by spoligotyping.

##### Post mortem examination

**Figure 4:**
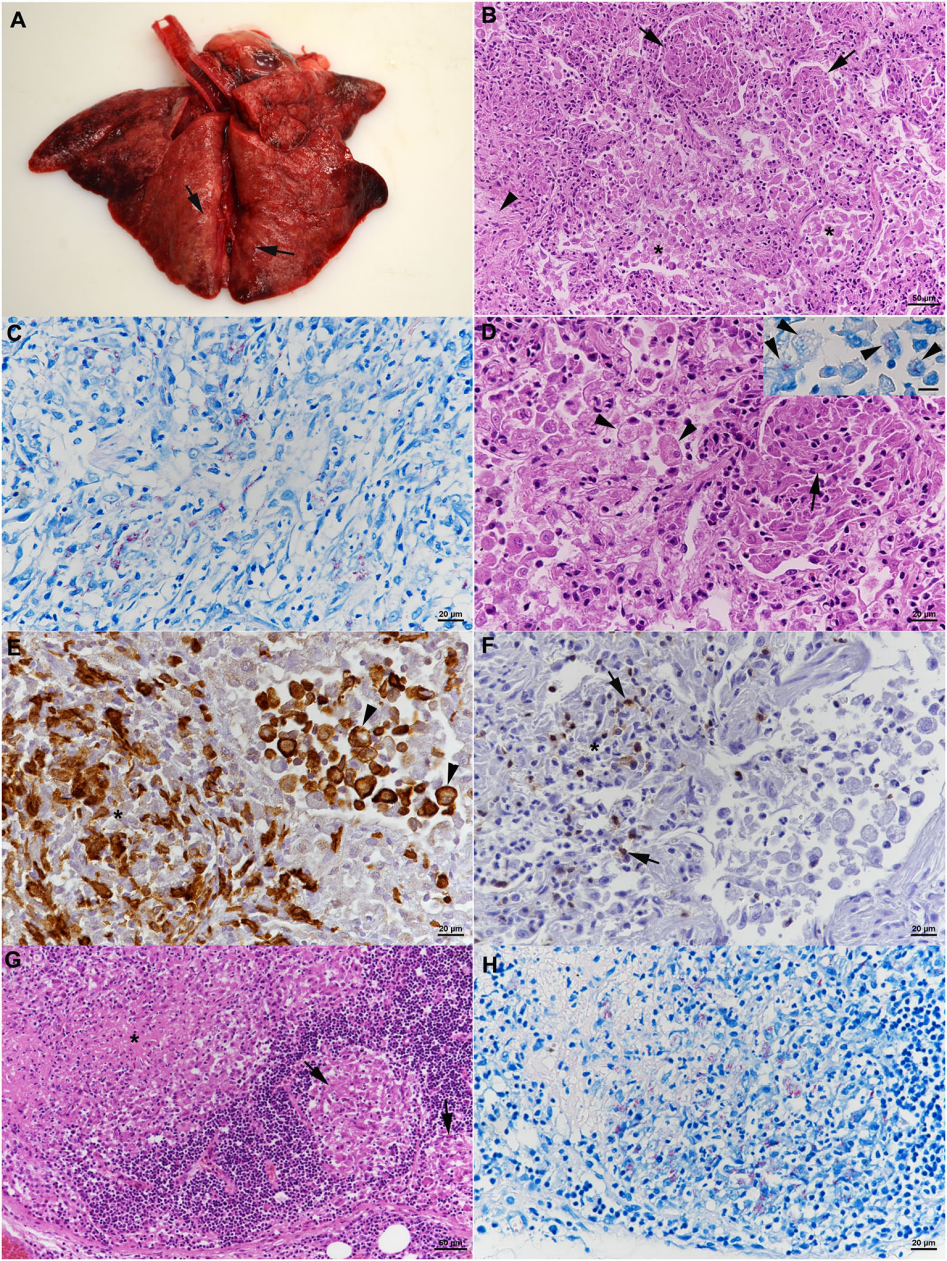
Case 2, histological features. **(A–F)** Lungs. **(A)** Gross picture of the lungs after exenteration. All lung lobes appear consolidated, with focal nodular thickening of the parenchyma (arrows). **(B)** Severe pneumonia with multiple granulomatous infiltrates (arrows) and focal areas of fibrosis (arrowhead). Alveoli often contain macrophages as well (*). HE stain. Bar = 50 μm. **(C)** Numerous macrophages and epithelioid cells contain individual or bundles of acid fast bacilli (AFB). Ziehl Neelsen (ZN) stain. Bar = 20 μm. **(D)** Closer view of granulomatous infiltrate (arrow) and alveoli that are filled with desquamed vacuolated alveolar macrophages/type II pneumocytes (arrowheads) that also contain AFB (inset; ZN stain). HE stain. Bars = 20 μm. **(E)** The granulomatous infiltrates (*) are dominated by Iba-1^+^ macrophages/epithelioid cells. Staining of cells in the alveolar lumina shows that the majority are macrophages (Iba-1^+^). Bar = 20 μm. **(F)** T cells (CD3^+^) are found intermingled in small numbers in the granulomatous infiltrates. **(G,H)** Mandibular lymph node. **(G)** Cortex with focal granulomatous infiltrates (arrows) and a larger infiltrate toward the medulla, with central necrosis (*). HE stain. Bar = 50 μm. **(H)** Numerous macrophages/epithelioid cells in the infiltrates contain AFB. ZN stain. Bar = 20 μm.

At necropsy, all lung lobes appeared consolidated, with nodular parenchyma and whitish to rose coloration (Figure 4A). Several lymph nodes (mandibular, mediastinal, mesenteric, pancreatic) were enlarged and exhibited a whitish cut surface. The histological examination revealed a severe chronic multifocal to coalescing pyogranulomatous pneumonia with focal areas of fibrosis (Figure 4B). Infiltrates were dominated by Iba-1^+^ epithelioid cells which were often shown to contain AFB (Figures 4C–E). T cells (CD3^+^) were found intermingled in small numbers (Figure 4F), MGCs were not observed (Figures 4D–F). In addition, multifocal alveolar distension by alveolar macrophages and type II pneumocytes, with intermingled neutrophils, was seen (Figures 4D,E); alveolar macrophages were found to also contain AFB (Figure 4D, inset). The grossly altered lymph nodes all exhibited a granulomatous lymphadenitis with the presence of variable amounts of intracellular AFB (Figures 4G,H), and occasional areas of necrosis, always without mineralization. Changes in the other organs comprised moderate lymphatic depletion in the spleen and a bilateral mild chronic lymphocyte-dominated interstitial nephritis. Granulomatous infiltrates were not observed.

#### Case 3

##### Clinical history (Figure 1C, Table 1A)

**Figure 5:**
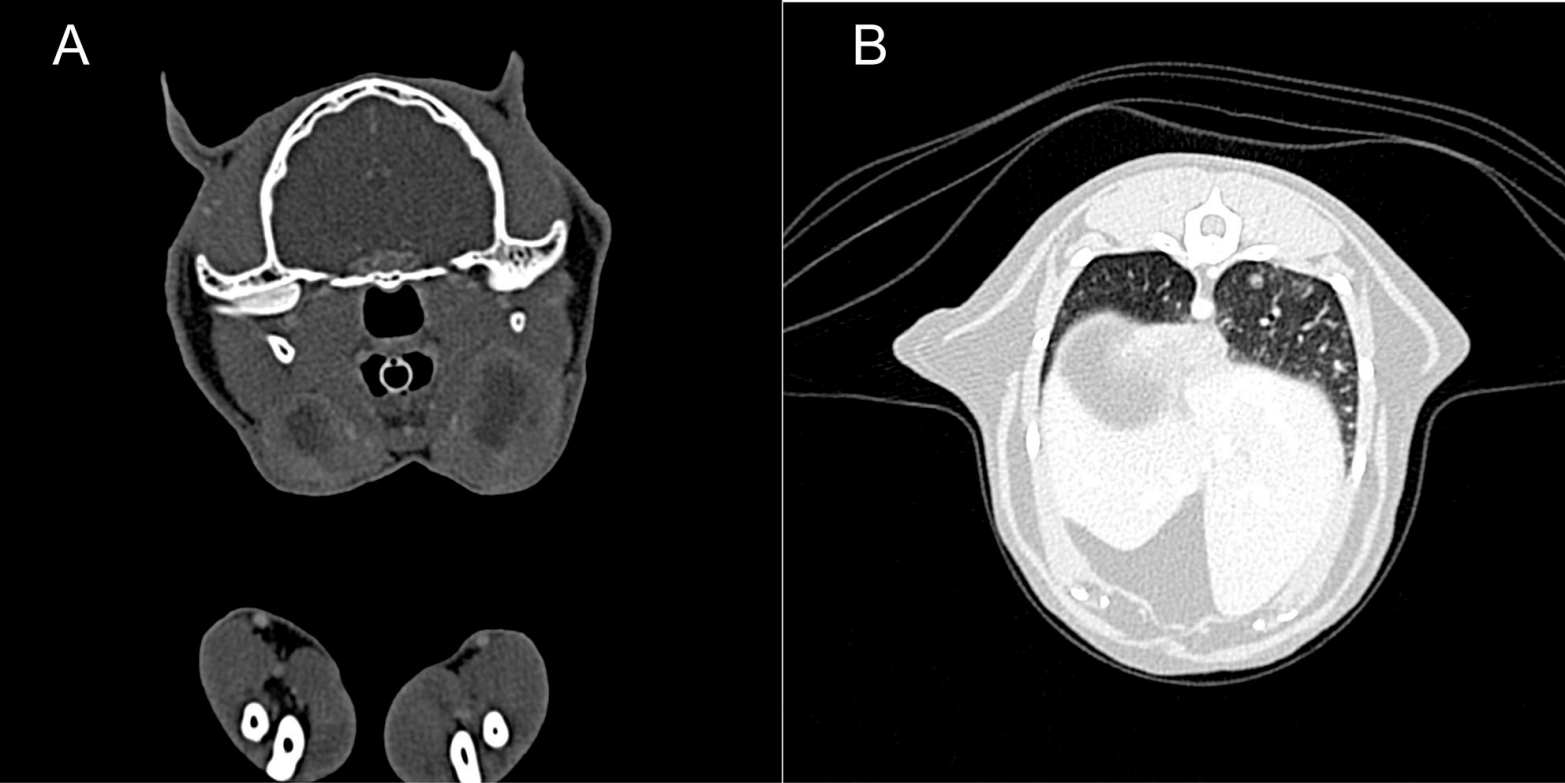
Case 3, diagnostic imaging. **(A)** Computed tomography of the head. The mandibular and retropharyngeal lymph nodes are markedly enlarged and show a heterogeneous and hypoattenuating center (up to 2 cm in long axis). **(B)** Computed tomography of the thorax. The pulmonary parenchyma shows a generalized miliary pattern with ground glass opacity and few soft tissue nodules (1–2 mm).

A 7-year-old male neutered domestic shorthair cat was presented to a veterinary practice for routine vaccination. The cat was free ranging and a known hunter. The general physical examination was unremarkable, apart from an enlargement of the right mandibular lymph node. Nine months later the cat was reassessed by the veterinarian, the lymph node had a size of ~8 mm in diameter and was hard on palpation. Routine hematology showed slight lymphocytosis, serum biochemistry was within normal limits. The cat was treated with a 6 days course of amoxicillin (12 mg/kg q12 h). Three weeks later the owner reported that the lymph node had resumed its normal size. One year later, the cat was presented to the veterinarian with enlargement of both mandibular lymph nodes. The owner reported apathetic behavior and abnormal respiratory sounds. The cat was in good general condition, no other lymph nodes were enlarged. The right mandibular lymph node measured about 20 mm, the left 15 mm in diameter. The animal was tested negative for FIV and FeLV infection. The cat was discharged with meloxicam (1 mg single injection) and a 10-days course of marbofloxacin (2 mg/kg PO q24 h). On presentation 3 weeks later, the mandibular lymph nodes were still enlarged, but reduced in size. A fine needle aspirate was collected, the cytological examination revealed a slight purulent lymphadenitis and reactive hyperplasia. Histopathology of biopsies from the mandibular lymph nodes revealed a severe chronic granulomatous to pyogranulomatous lymphadenitis; special stains (Periodic acid-Schiff reaction, ZN stain) did not detect fungal elements or AFB and a non-specific bacterial infection was suspected. Computed tomography revealed severe mandibular and retropharyngeal lymphadenopathy, multiple pulmonary nodules and a subcutaneous nodule adjacent to the left masseter (Figure 5A). The pulmonary changes were interpreted as neoplasia or granulomatous pneumonia (Figure 5B). The cat was discharged with an amoxicillin/clavulanic acid therapy for 10 days (15 mg/kg PO q12 h). Three months later, the skin lesions were surgically removed, and another biopsy taken from a mandibular lymph node. Both were submitted for histopathological examination and bacteriology. Heparinized blood was collected for IGRA. After conformation of MTBC infection, the therapy protocol was adjusted to azithromycin (12.5 mg/kg PO q24) and ciprofloxacin (3 mg/kg PO q24) for 6 months, and rifampicin (15 mg/kg PO q24) for two months (47). However, after 3 days of treatment the owners reported a severe deterioration of the cat's general condition, with apathy and anorexia, and decided to abort the medication. Three months later, the initially prescribed antimicrobial therapy (azithromycin and ciprofloxacin) was re-initiated for 20 days, because the mandibular lymph nodes were again enlarged. This therapy attempt did not result in consistent improvement of the lesions. The cat also developed an ulcerative wound (1 × 1.5 cm) at the left tarsus. During the next 5 months, no clinical improvement was seen, both the mandibular lymph nodes and the ulcerative wound had further increased in size (~2.5 cm diameter and 3 × 2.5 cm, respectively). Subsequently, the patient was lost to follow up.

##### Mycobacterial examination

The ZN staining of decontaminated material found curved acid-fast bacilli, and the real-time PCR for MTBC was positive. An HRM assay on biopsy material served for identification of the mycobacteria as *M. microti*. Mycobacterial culture was negative.

##### Histopathological examination

**Figure 6:**
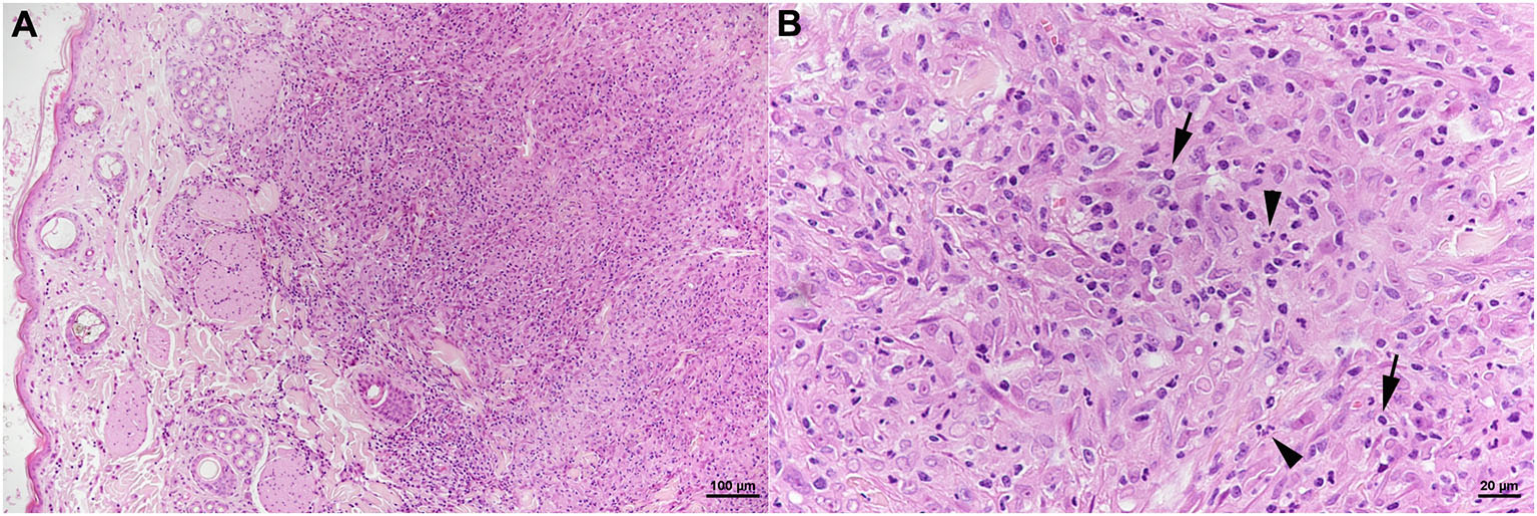
Case 3, histological features. Skin with focal extensive pyogranulomatous infiltration stretching from the superficial dermis **(A)**. **(B)** The infiltrate is dominated by epithelioid macrophages, intermingled with neutrophils (arrowheads) and fewer lymphocytes (arrows). HE stains. Bars = 100 μm **(A)** and 20 μm **(B)**.

The skin lesion was represented by a focal extensive pyogranulomatous infiltration stretching from the superficial dermis to the subcutaneous adipose tissue (Figure 6A). The infiltrate was dominated by epithelioid macrophages, intermingled with neutrophils and fewer lymphocytes (Figure 6B) which were almost exclusively T cells (CD3^+^). Together with B cells (CD45R^+^), the latter also formed small aggregates in the periphery of the infiltrate. The architecture of the mandibular lymph node was severely distorted by a similar pyogranulomatous infiltrate; follicles and T cell zones were restricted to remnants in the outer cortex. AFB were not identified in any of the lesions.

### Spoligotype Profiles

In 10 of the 11 cases, feline TB was identified by spoligotyping. All 10 cases presented the same spoligotype signature SB0118, characterized by the presence of spacers 37–38 (www.Mbovis.org).

### Results of the IGRA

Blood samples originating from two culture confirmed *M. microti* infected cats (Cases 1 and 3) reacted more intensely to BoPPD stimulation than AvPPD stimulation, indicating prior sensibilisation to MTBC antigens (Table 2). Fourteen healthy cats aged between 1 and 11 years were also assessed by the IGRA (negative controls). In the control group animals, stimulation with BoPPD and AvPPD did not induce the secretion of a measurable IFN-γ concentration. However, all negative control animals showed a distinct response to PMA/Ca (Table 2) indicating that viable cells had been recovered and successfully stimulated with the adapted protocol and PMA/Ca induced a strong cellular activation in cats.

**Table 2:** Results of the IFN-γ ELISA generated from two culture confirmed *Mycobacterium microti* infected cats and 14 healthy cats aged between one and 11 years are shown.

	**Mean IFN-γ response [Concentration C.I. (ng/ml)]**		
	**Bovine PPD**	**Avian PPD**	**PMA/Ca**
*M. microti*—positive			
1	0.50 (0.32–0.69)	<0.246	4.05 (3.73–4.33)
2	7.82 (5.41–10.18)	2.96 (2.29–3.56)	47.70 (15.46–79.79)
Control cats			
3	<0.246	<0.246	14.26 (8.30–20.20)
4	<0.246	<0.246	10.25 (6.48–3.39)
5	<0.246	<0.246	16.00 (10.30–22.20)
6	<0.246	<0.246	>60
7	<0.246	<0.246	0.54 (0.24–0.83)
8	<0.246	<0.246	1.98 (1.42–2.53)
9	<0.246	<0.246	0.60 (0.28–0.91)
10	<0.246	<0.246	4.06 (2.98–5.15)
11	<0.246	<0.246	4.78 (3.41–6.14)
12	<0.246	<0.246	4.69(3.36–6.01)
13	<0.246	<0.246	4.36 (3.08–5.37)
14	<0.246	<0.246	1.31 (0.89–1.72)
15	<0.246	<0.246	39.62 (10.46–74.79)
16	<0.246	<0.246	6.12 (2.98–9.36)

### Wild Rodents

A total of 346 rodents were trapped and culled; of these, 129 (37.3%) were *Apodemus* sp. and *Myodes glareolus* each, 86 (24.8%) *Arvicola scherman* and two (0.6%) *Microtus agrestis*. No common vole (*Microtus arvalis*) was trapped. None of the examined tissue pools yielded a positive result in the MTBC real-time PCR. Instead, several nontuberculous mycobacteria (NTM) and other AFB were found, as shown in Table 3. *M. lentiflavum* and *M. nebraskense* were the predominant species. Two pools showed a mixed culture with *M. nebraskense* and *M. lentiflavum*. All animals were examined histologically. None exhibited any evidence of granulomatous inflammatory lesions in any examined tissue.

**Table 3:** Non-tuberculous mycobacteria and other acid-fast bacilli isolated from tissue pools of wild mice.

**Isolated mycobacteria (NTM)**	**Number of pools (total 116)**	**Wild mice species**
*M. lentiflavum*	9	14 My/6 Ap/7 Ar^†^
*M. nebraskense*	6	9 My/3 Ap/6 Ar
*M. nebraskense*/*M. lentiflavum*	2	6 Ar
*M. vaccae*	3	9 Ar
*M. septicum*	1	1 My/2 Ar
*M. paragordonae*	1	3 Ar
Other isolated acid fast bacilli		
*Nocardia veterana*	1	1 Ar
*Streptomyces* sp.	1	1 Ar

† *My, Myodes glareolus; Ap, Apodemus sp.; Ar, Arvicola scherman*.

## Discussion

We report on a series of 11 natural cases of feline tuberculosis caused by *M. microti* that occurred between 2014 and 2019 in seven different cantons in Switzerland. Due to the unspecific symptoms presented by the patients, the chronicity of the disease and the often paucibacillary nature of the lesions, this number may not reflect the real extent of the problem and numerous cases may undergo undetected (23, 48). According to the veterinary personnel involved in the cases, the majority presented with localized or generalized lymphadenopathy, always involving one or both mandibular lymph nodes, and often the pre-scapular or popliteal lymph nodes. Subcutaneous nodules are frequent on the face and/or limbs, in most cases these remain localized and eventually ulcerate (48, 49).

There are speculations about the natural transmission of the bacteria, and involvement of the mandibular lymph nodes may suggest the oral infection route. Transmission from small rodents, the maintenance hosts of *M. microti*, seems obvious for infected cats that are in general reported as free roaming. In the UK 19% of mycobacterial infections in cats are caused by *M. microti* (50). In Switzerland *M. microti* infections have so far been reported from New World Camelids (15, 16), wild boars (46), and a cat (23), indicating that a rather wide range of animals is susceptible to infections with the vole bacillus. In a former study in the UK, *M. microti* tuberculosis was found in up to 13.2% of trapped *Microtus agrestis* in a specific region (31). The disease has previously also been reported in bank voles (Myodes glareolus), wood mice (Apodemus sylvaticus), and shrews (Sorex araneus) in the UK (12).

Interestingly, six animals (four cats, one Ilama, one wild boar) diagnosed with *M. microti* at our laboratory between 2011 and 2017 originated from the same region in Switzerland, the Gantrisch Nature Park (414 km^2^). To date, the spoligotype profile SB0118 is the only *M. microti* profile detected in Switzerland and it was isolated from a large host range including domestic cats, South American camelids, wild boars and captive gibbons (38). The same signature is also known in the international spoligotyping database SpolDB4 as ST 539 and has been described in numerous animal species as well as humans in France, the Netherlands and the UK (51, 52).

This prompted us to search for a common source of these infections, and we trapped and tested a substantial number of common wild rodents in the region. Surprisingly, none of the animals in this study tested positive for MTBC by bacteriological culture and real-time PCR. Although pooling of three wild rodents for molecular and culture-based detection of TB was a weakness of the investigation due to a certain dilution effect, the wild rodents (*n* = 346) were inspected individually for TB-compatible lesions macroscopically and histologically. From all animals, liver, spleen, lungs, mandibular, and mesenteric lymph nodes were collected and histologically evaluated. None exhibited any evidence of granulomatous inflammatory processes. There are different potential explanations for these results; (i) *M. microti* has a very low prevalence in Switzerland, either generally or due to natural annual fluctuations; (ii) wild small rodents are not the source of infection for cats in Switzerland, yet no other possible species has been scientifically disclosed; (iii) the wild rodents examined in the study are in the majority not a reservoir for *M. microti*. For *Microtus agrestis*, which has been confirmed as reservoir for *M. microti* in the UK, to be excluded as reservoir in Switzerland, the number of tested animals (*n* = 2) was too low. Typically, this species is more often found in the eastern than in the western part of Switzerland (53) where the mousetraps were set up. However, based on the present findings the remaining mouse species, *Myodes glareolus, Apodemus* sp., and *Arvicola scherman*, are not likely to play a role in the maintenance of *M. microti* in the investigated region. In addition, no *Microtus arvalis* were trapped and investigated for the presence of *M. microti* infection. Therefore, the potential role of this mouse species remains unknown.

Non-tuberculous mycobacteria were isolated from the analyzed wild rodents, but infection was not associated with any pathological processes. To date, little is known about the prevalence of NTM infections in wild rodent species, their pathogenicity and the significance of these host species in transmission and environmental contamination (54, 55). Hence the relevance of isolating opportunistic pathogens such as *M. nebraskense* and *M. lentiflavum* from wildlife is unclear. Only recently, a study in Spain tried to identify small mammals, mainly *Apodemus* sp., as the source of NTM infection in cattle which can lead to cross-reactive responses that can interfere with the diagnosis of tuberculosis (56). The authors identified six different NTMs (*M. intracellulare, M. avium* subsp. *paratuberculosis, M. gordonae, M. celatum, M. fortuitum*, and a not determined *Mycobacterium* sp.) with a prevalence of 6.5%. It is not clear whether these mycobacteria are able to colonize the mice or only pass through the animals. However, the movement of these animals leads to further spread (56) and may be problematic for livestock animals. The virulence of NTMs, including *M. lentiflavum*, in mice has been compared to the virulence of *M. intracellulare* and reported as lower (57). However, the opportunistic pathogen *M. nebraskense* was recently described as an animal pathogen, causing skin lesions in cats and dogs (58, 59).

All three feline cases presented here were free-ranging and known mouse hunters; all exhibited the initial lesions in the mandibular lymph nodes and, in two cases, the skin, representing the typical fight and bite sites of *M. microti* lesions (31). However, since we did not find evidence that the examined mouse species are a potential source of infection, which leaves the question open how the cats contracted the disease. A cat to cat transmission is possible, however not regarded as a main route. An increased risk of infection due to immunosuppression can be ruled out, since all three cats were tested negative for FIV and FeLV. Due to the potential zoonotic risk related to MTBC members, rapid and accurate identification of the mycobacterial species causing disease in companion animals such as cats is crucial. The testing of animals that live in close contact with vulnerable individuals such as children, elderly people or immunocompromised patients is hence considered necessary by public health authorities. As soon as the diagnosis of MTBC is made by a reference laboratory, the exclusion of *M. bovis* and *M. caprae* is of primary importance for veterinary authorities, and the exclusion of *M. tuberculosis* is critical for public health organizations. To date, molecular testing based on cultured bacteria is still the gold standard for this kind of differentiation. Because of the fastidious nature of *M. microti* and the extremely slow growth rate of specific animal strains, this differentiation can take several months or remain incomplete in cases where culturing of the mycobacterium is not possible. Based on the published data it has to be assumed that a large proportion of *M. microti* infections remain culture negative, even if the incubation time is prolonged to 18 weeks (13). A new HRM assay allows the detection of MTBC bacilli at species level in biopsy material (33, 34), making a fast species identification possible without the long culture period. This assay was successfully used for immediate species identification in Case 3 and is especially useful as shortcut diagnostics in presumptive *M. microti* infections. However, it requires invasive biopsy excision under general anesthesia and is therefore only suitable for animals with clinical signs.

Non-invasive diagnostics are needed for asymptomatic cases and contact animals. Rhodes et al. described a promising *in vivo* blood test for the diagnosis of tuberculosis in cats, by cell-based IFN-γ testing (36, 40). In the present study, the feline adapted IGRA detected both *M. microti* infected cats that were tested and yielded a negative result for the 12 healthy control cats. The relatively low response of Case 1 to BoPPD stimulation may be explained by the late stage of the disease since the blood sample was collected prior to euthanasia, when the general condition and possibly the responsiveness of the immune system of the patient were severely reduced, e.g., T cells involved in the type IV hypersensitivity reaction (60). Although fluctuations of the IFN-γ response *in vitro*, and a general decrease of cell-mediated immunity as the disease develops have been described, e.g., in humans and cattle, only limited information is available about the fluctuation of IGRA responses during feline tuberculosis (40).

The histopathological examination of the lesions revealed granulomatous to pyogranulomatous inflammatory processes that were dominated by often large epithelioid cells but did not contain MGCs. The absence of MGCs is a common feature in feline TB that can in paucibacillary lesions at least partially be explained by the low number of bacilli, since MGC formation is induced by mycobacteria and then maintained in an autocrine fashion by the MGCs themselves (61). On the other hand, MGCs are generally only found in granulomas, defined layered structures where MGCs develop from fusion of macrophages in close proximity to the tightly interdigitating epithelioid cells (62). However, granuloma formation, in the sense of the structured epithelioid granuloma in TB (62), was not seen in the present cases, similar to previous descriptions of *M. microti* induced lesions (23, 48, 50). The lack of granuloma formation maybe due to the attenuated nature of *M. microti* as a consequence of RD1 deletion (27), since previous studies have shown that mycobacteria lacking the ESX-1 secretion system encoded by RD1 are associated with poor granuloma formation (62–64). However, host specific differences in response to the bacterium must also play a role since *M. microti* tuberculous lesions in field voles (*Microtus agrestis*), the maintenance hosts of the bacterium identified in the UK, were typical granulomas (31). Similar to the situation in field voles, the presence of lesions in the skin and/or mandibular lymph nodes in the feline cases suggests infection through wounds in skin and oral cavity, whereas the consistent involvement of the lungs is most likely a consequence of bacteremia that would also be the basis for involvement of further lymph nodes, spleen, and other organs (31). The lesions in the lungs, however, provide strong evidence of bacterial shedding, either as a consequence of inflammatory processes that break into airways, or by infection of alveolar macrophages that are part of the exudate (31). The feline case series reported here also shows the morphological versatility of the lesions induced by *M. microti*. In light of the absence of MGCs and AFB and the often desquamative component of the pulmonary lesions, a high level of alertness is required from the diagnostic pathologist, which in a bTB free country like Switzerland, with low prevalence of human tuberculosis, is a challenge. The clinical history, and in particular the unexplained enlargement of individual palpable lymph nodes, might be the best indicator.

In the absence of a validated and certified ante mortem diagnostic tool for cats, the IFN-γ assay provides important information for the veterinary practitioner. Despite the possible cross-reactivity with NTM, the use of BoPPD was preferred to the more specific peptide cocktails, e.g., early secretory antigen target-6 (ESAT-6) or a 10-kDa culture filtrate protein (CFP-10) in the present study, for various reasons (65). First of all, the possible cross-reactivity with defined NTM in cats is yet to be proven and the actual sensitization or infection status in this species is largely unknown. Secondly, because of the actual epidemiological situation in Switzerland, the main causative agent for feline tuberculosis is apparently *M. microti*. Because of the peculiar RD1 deletion present in this MTBC member, a previous sensitization or an infection with *M. microti* would not be detected by the use of ESAT-6 and CFP-10 as stimulants (36). The components of PPD are poorly characterized and difficult to standardize, therefore antigens specific to the Mycobacterium tuberculosis complex have been investigated. Although an increase in specificity has been observed with specific antigen cocktails including ESAT-6, CFP-10, and Rv-3615c in cattle, the sensitivity achieved using such stimulants is still lower than that obtained with the bovine PPD (66–68). Nevertheless, the test specificity when other mycobacterioses are suspected has still to be investigated. A positive IFN-γ assay cannot replace anamnestic, radiological, and histopathological findings that are fundamental for the diagnosis of feline tuberculosis. However, it is a valuable screening method that only requires minimally invasive approaches by veterinary practitioners confronted with suspected feline tuberculosis cases, since it can guide the subsequent specific diagnostic approaches.

## Conclusions

Feline tuberculosis is rarely diagnosed in Switzerland, and the current study presents the particular diagnostic challenge associated with *M. microti* infections in cats. However, tuberculosis should always be considered a differential diagnosis in cats with therapy-resistant lymphadenopathy and recurring skin nodules. The combination of advanced imaging, histopathological, immunological, and molecular analysis enables a fast and highly sensitive diagnostic approach. The feline adapted IGRA is a promising screening tool for presumptive feline tuberculosis cases. The present study failed to identify the source of infection for cats in Switzerland, though the distribution of lesions suggests that these are prey animals.

## Data Availability Statement

The original contributions presented in the study are included in the article/supplementary materials, further inquiries can be directed to the corresponding author.

## Ethics Statement

This project was performed in accordance with the Swiss Animal Welfare Act (SR 455) and the specific regulations of the Cantons of Bern and Fribourg (permit number BE145/16).

## Author Contributions

GG, SP, and PL designed and coordinated the study. PL trapped and examined the mice. FO, MD, BW, MS, SH, and KK provided the clinical data. GG and UF performed the experiments. AK carried out the histopathological examinations. SP and GG analyzed the data and drafted the paper. All authors reviewed and approved the final manuscript.

## Conflict of Interest

The authors declare that the research was conducted in the absence of any commercial or financial relationships that could be construed as a potential conflict of interest.
